# Germline recessive mutations in *PI4KA* are associated with perisylvian polymicrogyria, cerebellar hypoplasia and arthrogryposis

**DOI:** 10.1093/hmg/ddv117

**Published:** 2015-04-08

**Authors:** Alistair T. Pagnamenta, Malcolm F. Howard, Eva Wisniewski, Niko Popitsch, Samantha J.L. Knight, David A. Keays, Gerardine Quaghebeur, Helen Cox, Phillip Cox, Tamas Balla, Jenny C. Taylor, Usha Kini

**Affiliations:** 1 National Institute for Health Research Biomedical Research Centre, Wellcome Trust Centre for Human Genetics, University of Oxford, Oxford OX3 7BN, UK,; 2 Section on Molecular Signal Transduction, Program for Developmental Neuroscience, Eunice Kennedy Shriver NICHD, National Institutes of Health, Bethesda, MD 20892, USA,; 3 Institute of Molecular Pathology, Vienna 1030, Austria,; 4 Department of Neuroradiology,; 5 Department of Clinical Genetics, Oxford University Hospitals NHS Trust, Oxford OX3 9DU, UK,; 6 West Midlands Regional Clinical Genetics Service, Clinical Genetics Unit and; 7 Department of Histopathology, Birmingham Women’s Hospital NHS Foundation Trust, Birmingham B15 2TG, UK

## Abstract

Polymicrogyria (PMG) is a structural brain abnormality involving the cerebral cortex that results from impaired neuronal migration and although several genes have been implicated, many cases remain unsolved. In this study, exome sequencing in a family where three fetuses had all been diagnosed with PMG and cerebellar hypoplasia allowed us to identify regions of the genome for which both chromosomes were shared identical-by-descent, reducing the search space for causative variants to 8.6% of the genome. In these regions, the only plausibly pathogenic mutations were compound heterozygous variants in *PI4KA*, which Sanger sequencing confirmed segregated consistent with autosomal recessive inheritance. The paternally transmitted variant predicted a premature stop mutation (c.2386C>T; p.R796X), whereas the maternally transmitted variant predicted a missense substitution (c.5560G>A; p.D1854N) at a conserved residue within the catalytic domain. Functional studies using expressed wild-type or mutant PI4KA enzyme confirmed the importance of p.D1854 for kinase activity. Our results emphasize the importance of phosphoinositide signalling in early brain development.

## Introduction

Polymicrogyria (PMG) is a malformation of the cerebral cortex that results from abnormal neuronal migration and organization. It may be missed prenatally as abnormal gyral formation does not become obvious on antenatal scans before 24 weeks gestation and routine detailed antenatal ultrasound scans take place usually around 20 weeks. Fetal magnetic resonance imaging (MRI) is helpful in confirming the diagnosis. Postnatally, it is most commonly detected using MRI and is characterized by an increased number of small gyri and disruption to the normal pattern of cortical lamination. The clinical presentation is variable often including learning disability, epilepsy and other neurological symptoms such as hemiparesis (1); some individuals may even be asymptomatic.

Although there has long been evidence that the causes of some forms of PMG are environmental (2,3), the familial recurrence often observed (4,5) and the chromosomal abnormalities associated with PMG (6) suggests that genetics also plays an important etiological role. The first gene to be implicated in non-syndromic PMG, *GPR56*, was identified in 2004 using microsatellite markers and a linkage approach and shown to result in the specific phenotype of bilateral frontoparietal PMG (7). Two PMG-associated genes (*EOMES* and *NHEJ1*) were uncovered by cloning the breakpoints of balanced translocations (8,9). Many of the more recently identified PMG candidate genes were found using a combination of autozygosity mapping and exome sequencing (10,11). However, although there are now over 20 PMG genes known (Supplementary Material, Table S1), many cases remain unexplained.

PMG has also been associated with 22q11.2 and 1p36 deletion syndromes (12–14). Mutations in *SNAP29*, a gene which lies within the 22q11.2 deletion region, cause the autosomal recessive condition CEDNIK, which comprises cerebral dysgenesis, neuropathy, ichthyosis and keratoderma and can include PMG (15). One study of note analysed *SNAP29* in patients with atypical clinical presentations of 22q11.2 deletion syndrome and found hemizygous mutations in 4 of 17 cases, of which two had PMG and microcephaly (16).

A number of recent studies have identified a new category of *de novo* activating mutations in the *PI3K-AKT-mTOR* signalling pathway which can lead to megalencephaly (17,18), with or without PMG (19). Such mutations are often found to have occurred postzygotically and hence are detected in a mosaic state. PI3K-AKT-mTOR signalling is an important regulator of cellular growth and constitutive activation of this pathway is an important oncogenic mechanism (20).

As well as being used in research to help identify novel disease genes, exome sequencing has also met with great success in clinical genetics settings and recent studies have reported a diagnostic yield of 25–32% across a wide range of conditions (21–25). Fetal samples are often underrepresented in this type of exome sequencing study which may be either due to the difficulties in obtaining appropriate consent or the lack of sufficient good-quality DNA sample from the fetus. In this study, exome sequencing was performed on a couple and from samples from their three affected fetuses, all of whom had been terminated on account of multiple congenital abnormalities that included PMG. Using identity-by-descent filtering, we identified compound heterozygous mutations in a phosphatidylinositol kinase gene, which co-segregate with the disease and which were shown to functionally impair enzymatic activity.

## Results

### Clinical features of fetuses with *PI4KA* mutations

**Figure 1.**
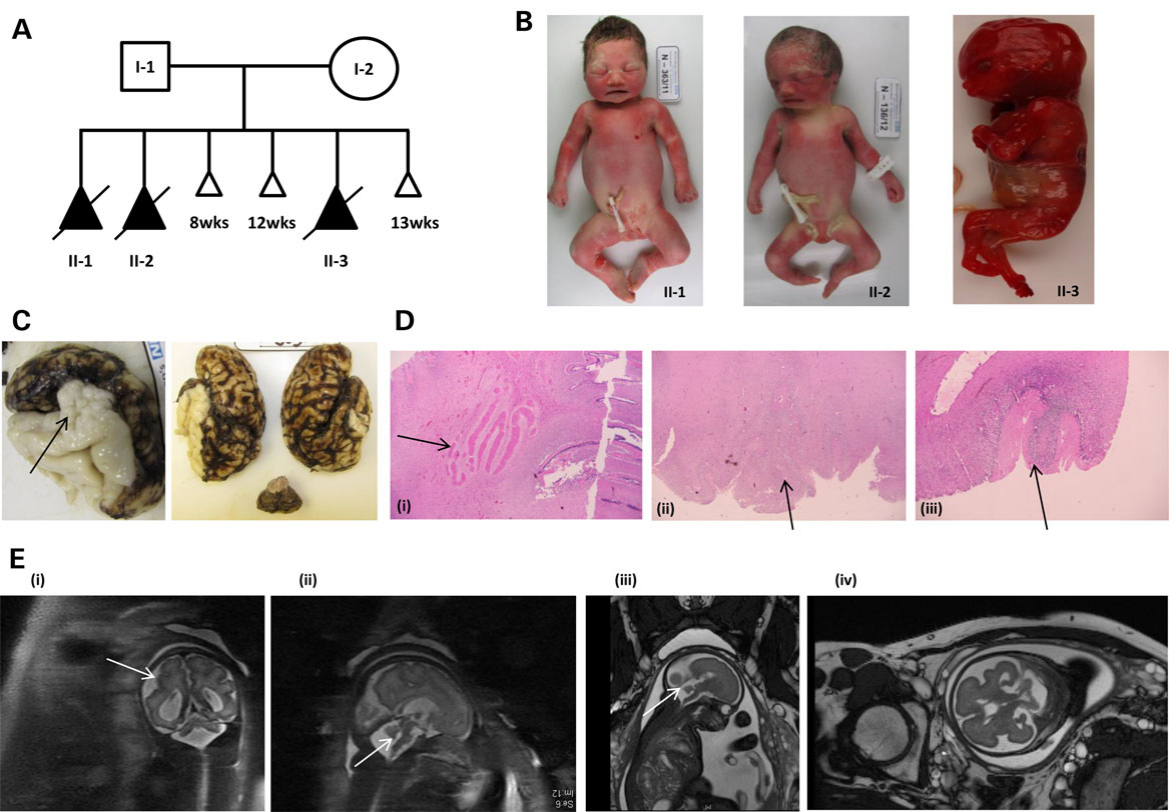
Pedigree, photographs, histology and MRI images of affected fetuses. (**A**) Pedigree showing three terminations of pregnancies (II-1 at 34 weeks, II-2 at 28 weeks and II-3 at 16 weeks) and three miscarriages. Shading indicates severe congenital abnormalities which were identified initially by prenatal ultrasound. WES was performed on the five individuals labelled, whereas SNP arrays were only performed on I-1, I-2 and II-2. (**B**) Photographs showing three female fetuses, all with arthrogryposis and micrognathia. (**C**) Macroscopic appearance of fixed brain of fetus II-1 with PMG and small cerebellum. (**D**) Histopathological appearances of the brain. (i) Dysplasia of the cerebellar dentate nucleus. The nucleus is irregular and fragmented rather than forming a single continuous undulating ribbon. ((ii) and (iii)) PMG of the cerebral cortex—abnormal folding of the cortex which is disorganised with fusion of the small cortical folds in II-1 and II-2, respectively. (**E**) Fetal MRI scans ((i) and (ii)) showing PMG and small cerebellum in II-1 at 34 weeks, ((iii) and (iv)) showing small cerebellum and delayed sulcation in II-2 at 28 weeks. Arrows indicate the regions where PMG and cerebellar atrophy are most apparent.

The three female fetuses were all diagnosed *in utero* with multiple congenital abnormalities and the pregnancies were terminated at between 16 and 34 weeks (Fig. 1A–E). The post-mortem report highlighted that the fetuses had bilateral perisylvian PMG with other brain abnormalities such as cerebellar hypoplasia and abnormal olivary and dentate nucleus. All three fetuses were noted to have severe bilateral talipes, externally rotated hips and a flexed posture. The muscle histology was reported to be within normal limits. Other limb abnormalities included flexion deformity of the wrist and overlapping fingers. In II-2 and II-3, the overall weight was noted to be larger than expected for gestation (98th centile) and in II-3, the weight of the brain was particularly noted to be larger than expected. II-1 had a normal 46, XX karyotype and genetic testing of *GPR56* did not uncover any variants of predicted clinical significance. The unaffected parents are of European ancestry and have had three early miscarriages in addition to the fetuses described here (Fig. 1A). Clinical details are summarized in Table 1. The family was recruited into the ongoing Structural Brain Abnormalities and Learning Disabilities Study, which received UK ethics approval (12/WA/0001) and informed consent was obtained from both parents.

**Table 1.** Clinical summary of the three affected fetuses

	Fetus II-1 (34/40)	Fetus II-2 (28/40)	Fetus II-3 (16/40)
Post-mortem brain weight in grams (expected weight ± 1 SD^a^)	178 (308 ± 49)	176 (219 ± 52)	21.8 (17.0)
Post-mortem body weight in grams (expected weight ± 1 SD^a^)	2328 (2250 ± 200) 55th centile	1404 (1020 ± 340) 98th centile	138 (90)
PMG	Perisylvian (bilateral asymmetric)	Perisylvian (bilateral)	Perisylvian (bilateral)
Cerebellar vermis	Hypoplastic	Hypoplastic	Dysplastic
Other CNS abnormality	Small pons	Dysplastic dentate nuclei	Dysplastic dentate and olivary nuclei
Joint contractures	TEV (bilateral)	TEV (bilateral), flexed knees	TEV (bilateral), flexed knees and L. wrist
Renal pelviectasis	Bilateral	Unilateral	–
Lung development	Mild hypoplasia	Borderline hypoplasia and immaturity	Normal
Other	Asymmetric cerebral ventriculomegaly, micrognathia	Dolicocephaly, micrognathia, small tongue, absent uvula, overlap fingers 2 and 5	Dolicocephaly, micrognathia

TEV, talipes equinovarus.

^a^SD given where available (not available for 16-week gestation fetus).

### SNP array analysis

One small duplication of chrX:6 467 204–6 838 627 (hg19) was detected in II-2 which was maternally inherited. An X-linked disorder was not suspected for this family and as there were no RefSeq annotated genes within this region, this CNV was considered to be benign. No other likely etiological CNVs were identified. In addition, there were no significant regions of homozygosity identified, consistent with the parents (I-1 and I-2) reporting to be unrelated.

### Exome sequencing of PMG family

DNA samples from the parents and all three affected fetuses were submitted for exome sequencing. Across all five samples, the mean target coverage was 47.3–83.5×, with 91.1–93.8% of bases covered at greater than 10× (Supplementary Material, Table S1). We did not uncover any variant of likely clinical significance in our list of 22 known PMG candidate genes. The coding regions of these genes sum to a total of 53.8 kb and across the five samples, mean coverage of these regions ranged from 39.4× to 68.9×, with 88.9 to 92.8% of bases covered by at least 10 reads (gene-by-gene breakdowns are shown in Supplementary Material, Table S1 and Fig. S1).

**Figure 2.**
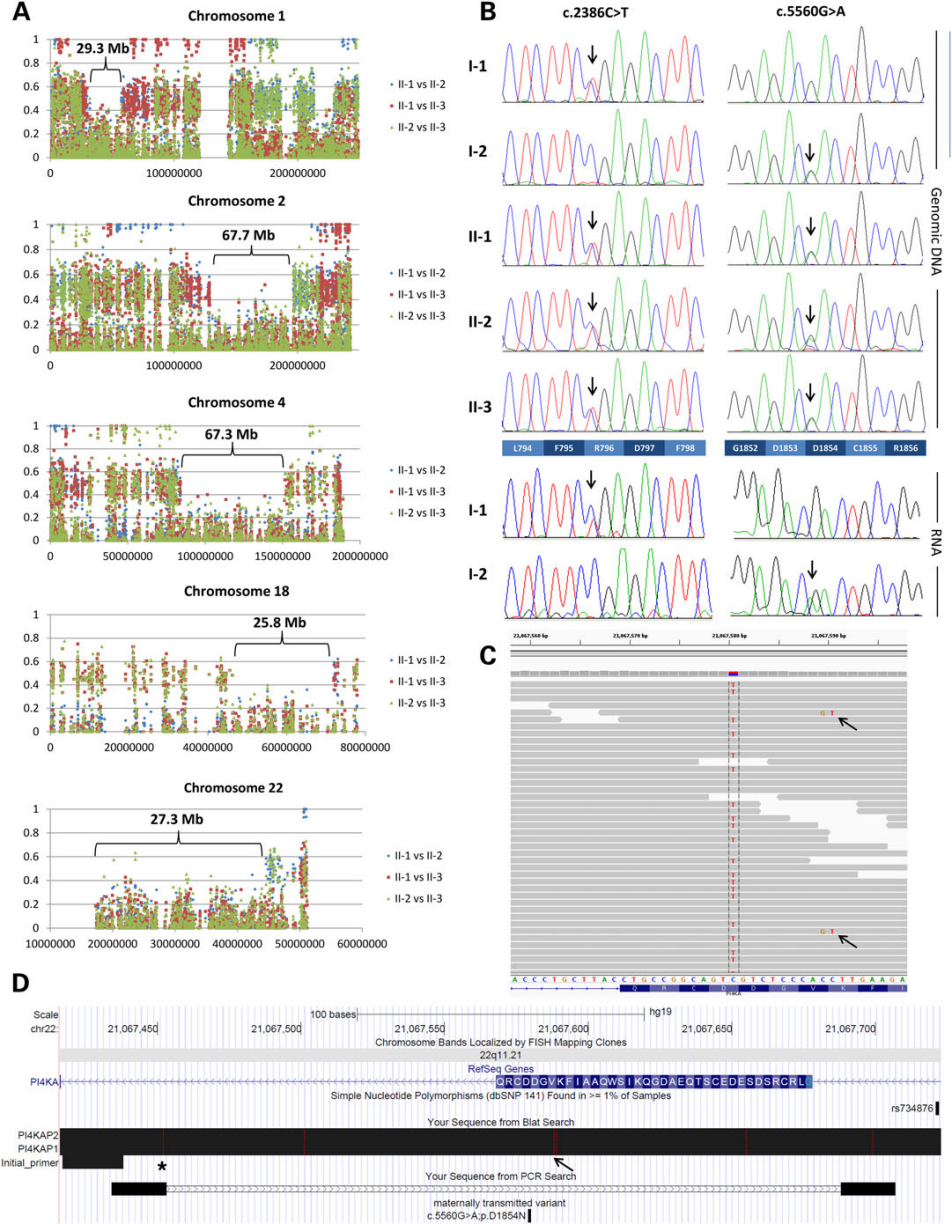
Genetic analysis. (**A**) Pairwise analysis for regions of identity-by-descent (IBD2). Absolute differences in allelic ratio are shown plotted against chromosome position. The largest five regions of shared IBD2 identified on chromosomes 1, 2, 4, 18 and 22 are labelled. In all cases, where IBD2 is detected in II-1 versus II-2 and II-1 versus II-3, IBD2 is also seen in II-2 versus II-3 (as expected). (**B**) Sanger sequencing confirming that the variants segregated consistent with recessive inheritance. Sequencing was performed in both directions. Both mutations were also detected in RNA from the respective parent. (**C**) Exome data from II-1 visualized in IGV. The arrows indicate two reads with an apparent CC>GT dinucleotide substitution, but which are really artefacts due to mismapping of pseudogene sequence. Reads that support the c.5560G>A variant (here shown on the +ve genomic strand as a C>T alteration) do not harbour the dinucleotide change, indicating that c.5560G>A is unlikely to be a mismapping artefact. (**D**) Image from the UCSC Genome Browser showing the positions of primers used to validate the missense variant. Sequences from *PI4KAP1* and *PI4KAP2* were mapped back to *PI4KA* using the BLAT tool such that any mismatches are shown by vertical red lines. The redesigned +ve strand primer has a 3′ mismatch with the pseudogene sequences, as shown by the asterisk. The −ve strand primer also has a mismatch in the middle. The position of the CC>GT dinucleotide mismatch is shown (by an arrow) at chr22:21 067 589-90. The window shown corresponds to chr22:21 067 417-21 067 723 (hg19).

After assessment of known genes, our *a priori* hypothesis was that any novel PMG disease gene would lie in a region of the genome where all three fetal samples shared both chromosomes identical-by-descent (IBD2). Unsure of how the relatively high error rate and uneven coverage of exome sequencing would influence a formal linkage analysis and motivated by the simple structure of the pedigree (Fig. 1A), we used a simple visual method that compares allelic ratios for high-quality variants. The allelic ratios for each SNV were obtained directly from the VCF file and absolute differences were plotted against genomic position. As sequencing coverage and accuracy increases, in regions of IBD2 absolute difference in allelic fraction should converge on zero (i.e. have the same genotype). There were nine genomic regions identified that all three fetuses shared IBD2 (Table 2), of which five were above 20 Mb in size (Fig. 2A). Of the 860 rare (<1% allele frequency) predicted deleterious variants detected within this family, 67 lay within IBD2 loci (Supplementary Material, Table S2). Among these variants were two mutations in *PI4KA*, identified within a 27.3-Mb segment of IBD2 on chromosome 22, that were shared by all three fetal samples. Even without the filter for IBD2 regions, these were the only rare predicted deleterious variants that fitted with the presumed autosomal recessive inheritance pattern. The first variant in *PI4KA* was a c.2386C>T mutation in exon 20 (NM_058004.3) that is predicted to result in premature termination of translation (p.R796X). The second variant, a c.5560G>A in exon 48 is predicted to result in a missense change (p.D1854N) in the kinase-binding domain of the protein (26). MutationTaster (0.999; see www.mutationtaster.org/), SIFT (0.003; see http://sift.jcvi.org/) and PolyPhen-2 (1.000; see http://genetics.bwh.harvard.edu/pph2/) all predicted the missense variant to be disease causing or damaging. Although neither variant was reported in 274 in-house genomes of mixed ancestry (27) or 500 Dutch genomes (28), both the nonsense and missense variants were later detected as a single occurrence in data from the Exome Aggregation Consortium (ExAC version 0.2; Cambridge, MA [accessed January 2015]) corresponding to allele frequencies of 1/122938 and 1/36994, respectively. Sanger sequencing confirmed that the two variants segregated consistent with autosomal recessive inheritance, with c.2386C>T inherited from the father (I-1) and c.5560G>A from the mother (I-2) (Fig. 2B).

**Table 2.** Regions of genome that are shared IBD2 between all three fetuses

Chromosome	Start (bp)	End (bp)	Size (bp)	Cumulative size (bp)	Cumulative % of autosomal genome
Chr1	29 652 068	58 939 634	29 287 566	29 287 566	1.01%
Chr1	213 140 398	229 599 225	16 458 827	45 746 393	1.58%
Chr2	128 936 000	196 663 997	67 727 997	113 474 390	3.93%
Chr4	83 857 109	151 161 470	67 304 361	180 778 751	6.26%
Chr9	139 371 405	141 213 431	1 842 026	182 620 777	6.32%
Chr16	89 345 408	90 354 753	1 009 345	183 630 122	6.36%
Chr18	46 196 998	71 989 909	25 792 911	209 423 033	7.25%
Chr20	40 714 524	52 185 814	11 471 290	220 894 323	7.65%
Chr22	17 280 822	44 586 522	27 305 700	248 200 023	8.59%

Coordinates given are based on hg19 and size of autosomal genome taken to be 2 888 135 837 bp.

### Analysis of *PI4KA* pseudogenes

Genetic analysis of *PI4KA* is complicated by the presence of two non-processed pseudogene partial copies, (*PI4KAP1* and *PI4KAP2*), each localized <1 Mb from *PI4KA*. Although homology with the pseudogene sequences does not extend to the region harbouring the c.2386C>T variant, when viewing the exome data for the c.5560G>A variant in IGV one sees a small percentage of reads generated from the pseudogene sequence that are mismapped onto *PI4KA*. These mismapping reads can be distinguished by the presence of an apparent dinucleotide substitution (chr22:21 067 589-90CC>GT) (Fig. 2C). None of the reads supporting the c.5560G>A mutation had this dinucleotide alteration suggesting that they had been mapped correctly. In addition, we repeated the Sanger sequencing using a new +ve strand primer that had been redesigned to have a mismatch with both pseudogene sequences at its 3′ end (Fig. 2D) and the c.5560G>A variant was still clearly visible (Supplementary Material, Fig. S2).

### RNA analysis

RNA was extracted from parental blood samples. Sanger sequencing of RT-PCR products showed that both mutations could also be detected at the RNA level (Fig. 2B). This provides further evidence (i) that the maternally inherited c.5560G>A mutation is present in the functional copy of the gene rather that in one of the pseudogenes and (ii) that the paternally inherited c.2386C>T mutation is not subjected to significant levels of nonsense mediated decay, at least in adult blood.

### Structural modelling

**Figure 3.**
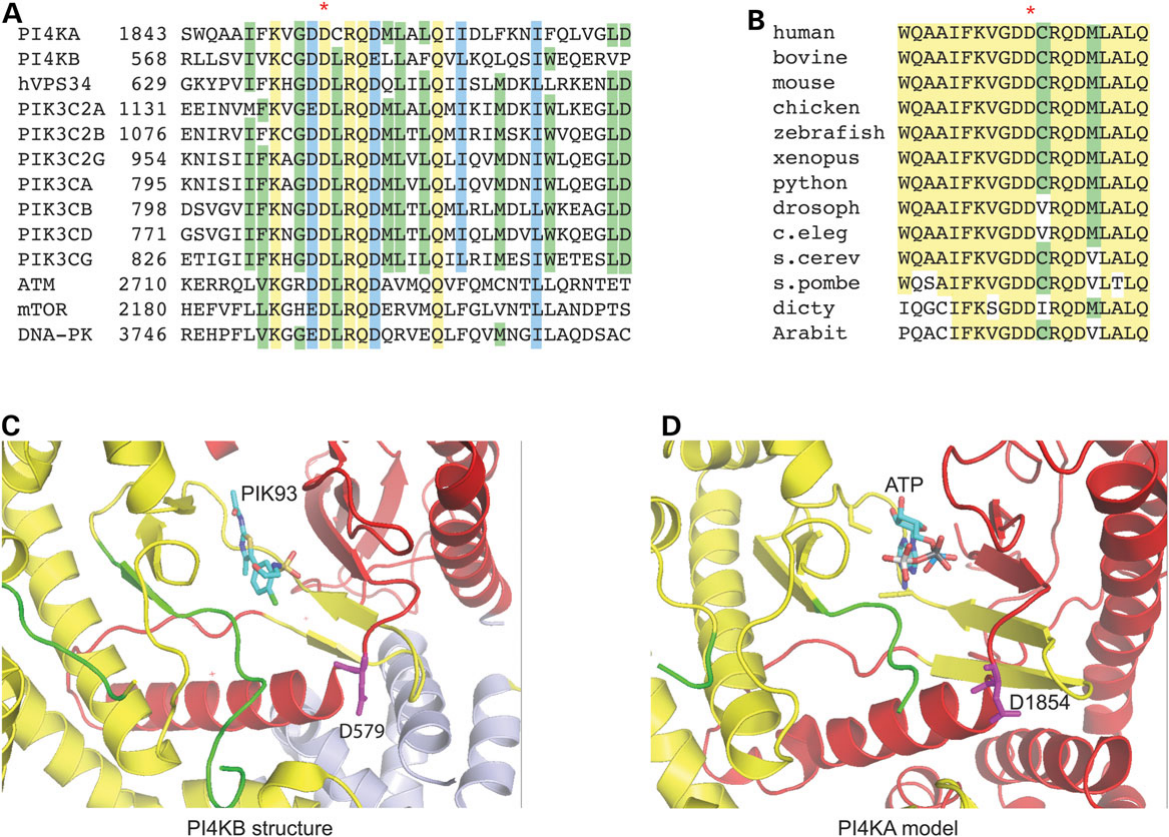
Amino acid conservation and structural modelling. (**A**) Sequence alignment of human type III PI 4-kinases, PI 3-kinases and PI kinase-related kinases within their catalytic domains. Highly conserved residues are highlighted in yellow and those with conservative substitutions with blue. Residues conserved between at least some of the groups are highlighted with green. The affected aspartate residue is labelled by a red asterisk. (**B**) Sequence alignment of orthologues of PI4KA from different eukaryotes. (**C**) The structure of the ATP-binding pocket of PI4KB with the ATP competitive inhibitor, PIK93 based on 4D0L (33). Red and yellow colours represent the C- and N-terminal halves of the catalytic site, respectively, while light blue is the N-terminal helical domain. Green indicates the activation loop. The conserved Aspartate residue (D579) is coloured magenta. (**D**) Model of the catalytic domain of PI4KA with ATP bound form (29) showing very similar features.

Sequence alignment of multiple PI4Ks and PI3Ks show that the Aspartic acid corresponding to the 1854 position in PI4KA is highly conserved among PI3Ks and type III PI4Ks and within PI4KA enzymes across evolution and even among the PI-kinase-related kinases (Fig. 3A and B) (29,30). Structural studies on PI 3- and 4-kinases (31–33) and modelling of the PI4KA catalytic domain (29) shows that while this residue does not make direct contact with the ATP molecule, it is critical to the proper folding of the ATP-binding pocket (Fig. 3C and D). Based on these findings, it was expected that a mutation at this position is not tolerated.

### Functional studies of kinase activity

**Figure 4.**
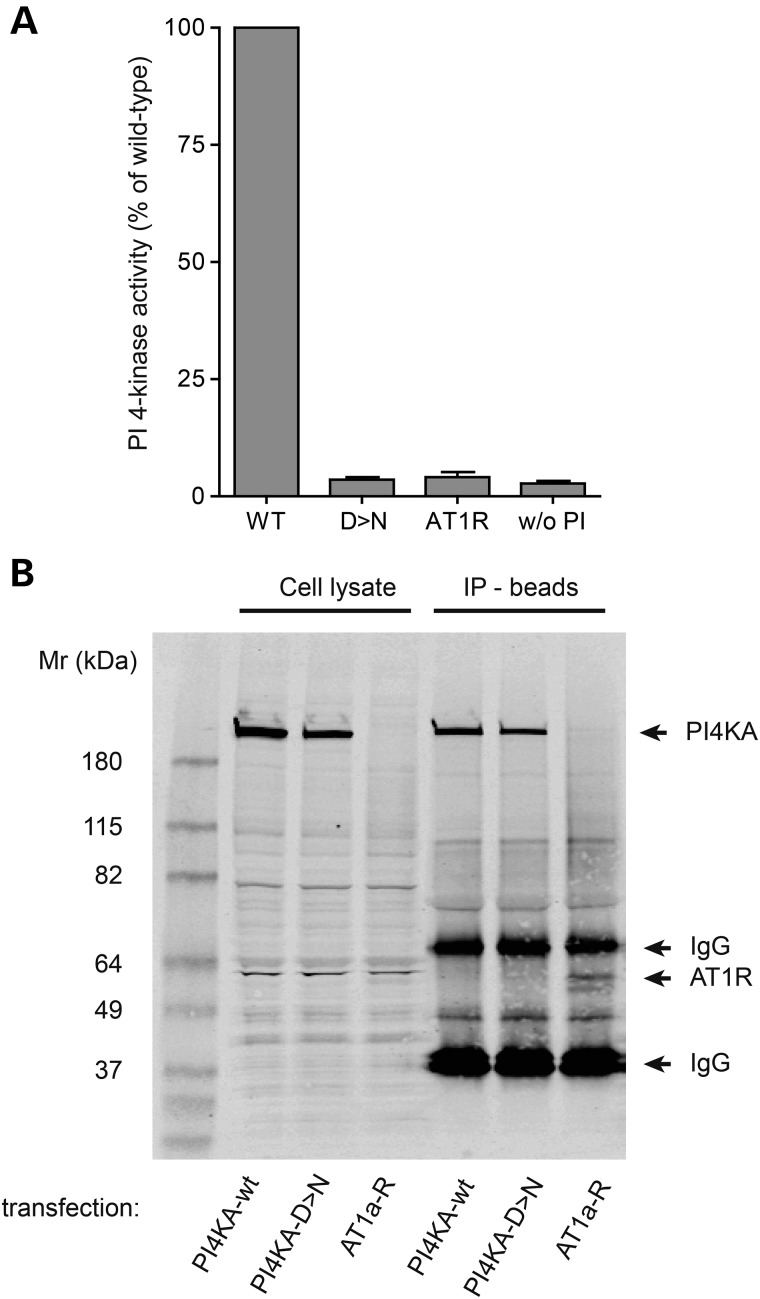
Functional studies of enzyme activity and protein stability. (**A**) *In vitro* PI 4-kinase activity assay performed on immunoprecipitated human PI4KA enzymes expressed in COS-7 cells. Wild-type or p.D1854N mutant enzymes were prepared and assayed with the ADP-GLO kinase assay as described under Materials and Methods. Activities were expressed as per cent of wild-type. Results are the means ± S.E.M from three independent kinase preparations. Note that the activity of the mutant enzyme is indistinguishable from the two controls, one measured from immunoprecipitates of AT1a angiotensin receptors (AT1R) and the other is assaying the wild-type enzyme without its substrate, PI (w/o PI). (**B**) Western blot analysis showing comparable expression of the wild-type and mutant PI4KA enzymes. Left lanes show the expression from the cell lysate and right lanes from the immunoprecipitates used for the kinase assay. Results shown are representative of three similar observations. The presence of the AT1 receptor in the lysates is not showing well in this exposure setting but the immature form of the receptor is visible (shown by the arrow) and the fully glycosylated receptor appears as a faint smear between the 82 and 115 kDa markers.

To determine the activity of the mutant enzyme, we generated the p.D1854N mutant form of the human PI4KA enzyme and compared its activity with the wild-type enzyme. N-terminally HA-tagged versions of the mutant and wild-type PI4KA were expressed in COS-7 cells and immunoprecipitated from the cell lysates with an anti-HA antibody. Cells transfected with an HA-tagged AT1 angiotensin receptor served as a control. Kinase activity was then measured on the beads using 0.3 mm ATP and 0.8 mm PI as substrates with the ADP-GLO kinase assay (34). As shown in Fig. 4A, the mutant enzyme had no measurable catalytic activity. Western blot analysis showed the presence of equal amounts of PI4KA enzyme in the immunoprecipitates (Fig. 4B).

## Discussion

In this study, exome sequencing was performed on a family comprising three fetuses with perisylvian PMG with cerebellar hypoplasia and both their parents. Having data for multiple affected fetuses allowed us to focus on regions of IBD2 and helped us identify compound heterozygous mutations in *PI4KA* as the only rare predicted deleterious variants that fitted the presumed autosomal recessive inheritance pattern. In addition to the co-segregation observed, the rarity of these variants in publically available controls databases, the predicted effect of the variants on translation or protein structure and the functional evidence confirming the effect on enzymatic activity all help support the pathogenic role of these variants. The high levels of PI lipids and PI4Ks in the brain (35–37) is suggestive of their importance in brain development (38) and indeed the role of the PI3K-AKT-mTOR signalling pathway in brain development is well established (39). Nevertheless, the identification of unrelated individuals with similar phenotypes and biallelic mutations in *PI4KA* would be the only definitive way of conclusively proving the disease association. Identification of such cases may be difficult due to the rarity and severity of the phenotype and the fact that fetal samples are typically underrepresented in clinical exome sequencing studies (25). In a recent exome sequencing study focusing on 30 samples where fetal structural abnormalities had been revealed by ultrasound, the diagnostic yield was only 10% (40), further highlighting the difficulties in identifying the causes of such severe phenotypes which present in fetal life.

*PI4KA* encodes phosphatidylinositol 4-kinase type IIIα that is involved with maintaining plasma membrane phosphatidylinositol 4,5-bisphosphate (PI4,5P_2_) pools (41). PI4,5P_2_ is an important substrate of both the PLC-InsP_3_-Ca^2+^ and the PI3K-AKT-mTOR signalling pathways (42). Variants in PI 3-kinases and their regulatory subunits, *PIK3CA*, *PIK3R2* as well as one of their downstream targets, *AKT3*, have all been implicated in PMG and associated brain malformation phenotypes (17–19,43). Variants in *AKT1* have been implicated in development disorders and overgrowth phenotypes (44,45). Mutations in this pathway are often activating mutations present in a mosaic state, although germline mutations have also been reported. Another recent study identified mutations in the PI(3,5)P_2_ phosphatase, *FIG4*, co-segregating with PMG in a consanguineous Moroccan pedigree, further strengthening the role of phosphoinositide signalling in brain development and showing that disease-causing mutations in this pathway do not always demonstrate dominant inheritance (10).

Two studies have reported the conditional inactivation of PI4KA in adult mice using tamoxifen-inducible whole body Cre strategy (41,46). In both studies, tamoxifen was administered via the oesophagus leading to death within 7 days due to severe gastrointestinal necrosis. Notably, tamoxifen exposure of the brain is limited under this regime and animals die before any brain symptoms can be observed. Acute inhibition of the enzyme by kinase inhibitors causes sudden death with symptoms indicating cardiovascular collapse (41). As these inhibitors were designed not to cross the blood brain barrier, no information is yet available on the effects of kinase inhibition on brain function. In the zebrafish model, downregulation of Pi4ka was described as causing multiple developmental abnormalities, affecting the brain, heart, trunk and most prominently causing loss of pectoral fins, similar defects to those seen in developing embryos treated with a PI3K inhibitor (38). Fly studies also showed that inactivation of the fly orthologue of PI4KA is lethal (47). Studies on yeast suggest that inactivation of the STT4 gene (the orthologue of PI4KA) results in lethality alleviated by osmotic stabilizers in some strains (48,49). In light of these observations, the severity of the phenotype seen in the family described here is not surprising given that one allele is a nonsense mutation, whereas the other results in undetectable kinase activity.

It is well known that increased rates of genotyping error can negatively impact on the success of linkage analysis (50) and so typically we perform linkage analysis on genotypes obtained from SNP arrays. Owing to limited DNA availability in this study, arrays could only be performed on one of the three fetuses. We therefore used the exome data to perform a simple visual search for genomic regions with high rates of shared genotype that are expected in regions of IBD2. In this way, we quickly reduced the search-space to 8.6% of the autosomal genome (in line with the theoretical expectation of 1/16), without requiring information about genetic distance, population allele frequency, disease frequency or that data are pruned to remove variants in linkage disequilibrium.

*PI4KA* is located in the region of chromosome 22 often deleted in 22q11.2 deletion syndrome, one of the most common clinically relevant microdeletions which has variable expressivity. It is of particular interest that PMG (particularly perisylvian) with or without cerebellar anomalies is a structural brain abnormality reported in individuals with 22q11.2 deletion. Robin *et al*. suggested that the PMG in 22q11.2 deletion syndrome may be a sequela of abnormal embryonic vascular development (13). Interestingly, they identified that in 8 of 24 patients, mild cerebellar hypoplasia and an enlarged cisterna magna were also present. All three fetuses in our study also showed cerebellar hypoplasia. We therefore hypothesized that the functionality of the remaining copy of *PI4KA* may account for the brain abnormalities seen in some patients with 22q11.2 deletion syndrome and carried out whole exome sequencing (data not shown) in one such individual with left-arm hemiparesis and diffuse PMG affecting the right hemisphere more than the left. In this patient, a 2.7 Mb deletion (18 894 835 to 21 561 514) had previously been detected by array-CGH (Agilent ISCA 60K array) during standard clinical genetic testing. No significant hemizygous changes were identified either in *PI4KA* or in *SNAP29*, another gene implicated in brain-related phenotypes associated with 22q11.2 deletion syndrome (16). The lack of variants in *PI4KA* in a single patient with 22q11.2 deletion and PMG does not rule out *PI4KA*'s involvement in this aspect of the variable phenotype as our analysis did not exclude that there may be functionally relevant non-coding variants in the remaining copy of this gene. It is also possible that there are other contributing factors that influence *PI4KA* function, such as DNA methylation which has previously been debated as a potential modifier in 22q11.2 syndrome (51,52).

In conclusion, we have identified a family in which compound heterozygous mutations in *PI4KA* co-segregate with structural brain abnormalities including perisylvian PMG and cerebellar hypoplasia. The data presented shed further light on the role of PIs and the importance of PI3K-AKT3-mTOR signalling in early brain development. Analysis of *PI4KA*, in a large case series of patients with 22q11.2 syndrome and PMG will test our hypothesis that this gene may be a determinant of the 22q11.2 deletion CNS phenotype. Exome sequencing of larger cohorts of fetuses with severe phenotypes such as that described here should not only help elucidate the phenotypic range associated with *PI4KA* mutations, but may also help identify novel disease genes where the presentation is too severe to be picked up in patient cohorts.

## Materials and Methods

### DNA extraction and SNP array analysis

Following informed consent from the parents, DNA was extracted from fetal tissue using the EZ1 DNA Tissue Kit (Qiagen). DNA from the parents was isolated from peripheral blood leucocytes using a magnetic bead method and the QIAsymphony automated extraction system (Qiagen). Samples from I-1, I-2 and II-2 were run on CytoSNP-12v2 arrays (Illumina) and analysed in Nexus Copy Number software v7.5 (BioDiscovery, Hawthorne, CA, USA).

### Exome sequencing

Whole Exome Sequencing was performed on the HiSeq2500 (Illumina) using the SeqCap EZ Human Exome Library v3.0 (NimbleGen). One hundred base pair reads were mapped to hs37d5 using Stampy version 1.0.22 (53) (www.well.ox.ac.uk/stampy). Variants were called with Platypus version 0.5.2 (54) (www.well.ox.ac.uk/platypus) with default settings except for minFlank = 0. Coverage was calculated on deduplicated BAM files using bedtools and CODOC (55). Variant calling was performed across all five BAM files simultaneously, producing a multi-sample VCF. The WES data from the family were extensively examined in Ingenuity Variant Analysis (version 3.1.20150207, www.ingenuity.com/products/variant-analysis) for any shared variants among the three fetuses. Although the *PI4KA* variants fitted a compound recessive model, we also searched for variants that fitted with other models such as simple recessive and *de novo* (with presumed parental germline mosaicism). For the population allele frequency filter, we used 1000 Genome Frequency (v5) and EVS (ESP6500 0.0.30). Our definition of ‘predicted deleterious' includes variants that introduce a frameshift, in-frame indels, stop codon changes, missense variants, splice site variants (up to two bases into an intron or which are predicted by MaxEntScan (56)) and variants which are reported in HGMD (version 2014.4). The final settings and other content versions used for filtering are available upon request.

### Analysis of identity-by-descent

Using customized scripts, the multi-sample VCF file was filtered for single-nucleotide substitutions that were flagged as PASS by the internal variant caller filter. Variants which lay in segmental duplications or repeat masked sequence were removed. Variants with <15× coverage in any of II-1, II-2 or II-3 were also eliminated. For the remaining 52 829 variants, the proportion of reads harbouring the variant was calculated. We then carried out pairwise comparisons for the three affected fetuses (i.e. II-1 versus II-2, II-1 versus II-3 and II-2 versus II-3) and plotted the absolute difference between the allelic ratio against genomic position. The median probe spacing was calculated to be 5.4 kb.

### Validation

PCR amplification was performed using the FastStart Taq DNA polymerase (Roche, Burgess Hill, UK). The stop gain mutation was amplified using the primers 5′-GTGGGCTCTGACCTCACC-3′ and 5′-ACCCAGTCTGAGTTTCTGAGA-3′, whereas the missense mutation was amplified using 5′-AGAAGCCCTAATTTACCCCGT-3′ and 5′-CTAACAGCGGCCTCTCTCC-3′. Due to the presence of two pseudogene copies of *PI4KA*, Sanger sequencing of the missense variant was repeated with a redesigned +ve strand primer 5′-CCGTGGCACCTGAACCATA-3′ (underlined bases designate mismatches with pseudogene copies). PCR products were purified using exonuclease I (NEB, Ipswich, MA, USA) and shrimp alkaline phosphatase (USB, Cleveland, OH, USA). Bidirectional Sanger sequencing was then performed using BigDye chemistry (Applied Biosystems, Foster City, CA, USA) and run on a 3730xl DNA Analyzer (Applied Biosystems).

### RNA analysis

Parental blood samples were collected in PAXgene blood RNA tubes and RNA was extracted using the PAXgene blood RNA Kit (Qiagen). cDNA was synthesized using the QuantiTect reverse transcription kit (Qiagen) which uses a mix of oligo-dT and random primers. PCR amplification and sequencing were performed as described above using the following primers: PI4KA-18F 5′-AGTTGTTTGTGCAGCTGGG-3′, PI4KA-21R 5′-GGGGACTTAGTGGCTATTTCA-3′, PI4KA-43F 5′-TTTATCTAGATGAAGAGGGCCAC-3′ and PI4KA-50R 5′-CGGGGATGCACTCGATCA-3′.

It should be noted that primer PI4KA-43F was designed specifically so it binds to an exon located outside the segmental duplication region. In all cases, the relevant RT −ve reactions were prepared and these did not result in any PCR amplification.

### Structural modelling

Structural modelling of PI4KA has been described previously (29).

### Measurement of enzymatic activity

COS-7 cells grown in 10 cm culture dishes were transfected with either the wild-type or mutant form of HA-tagged full-length human PI4KA or an HA-tagged rat AT1a angiotensin receptor (5 µg plasmid DNA per dish) using Lipofectamine Plus (Invitrogen) following the manufacturer's instructions. After 24 h, cells were washed with ice-cold PBS followed by lysis in 700 µl RIPA buffer (50 mm Tris pH 7.4, 150 mm NaCl, 1 mm EDTA, 0.25% deoxycholate, 1% NP40) freshly complemented with 1 mm Na-orthovanadate, 1 mm DTT, 10 µg/ml aprotinin, 5 µg/ml leupeptin and 1 µm AEBSF. Lysates were cleared with centrifugation (13 000*g*, 10 min, 4°C) and the lysates were incubated with 6 µl monoclonal anti-HA monoclonal antibody (Covance, clone HA.11, Princeton, NJ, USA) and 50 µl Protein G Sepharose overnight at 4°C. Samples were then washed five times with lysis buffer supplemented with 1 M LiCl and three times with kinase assay buffer. Beads were then resuspended in 200 µl kinase buffer (40 mm Tris, pH 7.5; 20 mm MgCl_2_; 1 mm EGTA; 0.2% Triton X-100; 0.1% BSA and 0.5 mm DTT) supplemented with 0.3 mm ultra-pure ATP and 0.8 mm phosphatidylinositol. After 1 h of incubation, beads were centrifuged and to the supernatant was added 200 µl ADP-GLO reagent (Promega) for a 40-min incubation at room temperature. At that point, 400 µl kinase-detection reagent was added and the samples were incubated for an additional 40 min in the dark. Reaction products were then read in a luminescence plate reader (Bertholds). Equal amounts of cell lysates (about 3% of total) and 40% of the eluates of the beads used for the kinase assays were loaded on 8–16% precast SDS gels (Novex, Life Technologies, Grand Island, NY, USA) and separated by gel electrophoresis. After transfer to nitrocellulose membranes and blocking with Odyssey blocking reagent, membranes were incubated with polyclonal rabbit HA antibody (1 : 400, Santa Cruz, Dallas, TX, USA) followed by incubation with an anti-rabbit-800 secondary antibody (1 : 10 000) from LiCor, Lincoln, NE, USA. Membranes were then read and analysed in an Odyssey infrared instrument (LiCor). Images were processed by Adobe Photoshop to adjust for best dynamic range but maintaining linearity.

## Note Added in Proofs

Since Advance Access publication we have been made aware of a study where a conventional KO of mouse PI4KA, which encodes PI4KIIIalpha, resulted in early embryonic lethality (57). However no data is available on how and what stage lethality occurred.

## Supplementary Material

Supplementary Material is available at *HMG* online.

## Funding

This was supported by the National Institute for Health Research (NIHR) Biomedical Research Centre Oxford with funding from the Department of Health's NIHR Biomedical Research Centre's funding scheme. The views expressed in this publication are those of the authors and not necessarily those of the Department of Health. The research of T.B. and E.W. was supported by the intramural research program of the Eunice Kennedy Shriver National Institute of Child Health and Human Development of the National Institutes of Health. Funding for the High-Throughput Genomics Group at the Wellcome Trust Centre for Human Genetics is from Wellcome Trust grant reference 090532/Z/09/Z and Medical Research Council Hub grant G0900747 91070). Funding to pay the Open Access publication charges for this article was provided by the Wellcome Trust.

## Supplementary Material

Supplementary Data
