# Supplementary material for: Germline recessive mutations in PI4KA are associated with perisylvian polymicrogyria, cerebellar hypoplasia and arthrogryposis
Source: Hum Mol Genet. 2015 Apr 8;24(13):3732–41. doi: 10.1093/hmg/ddv117 (PMC4459391; doi:10.1093/hmg/ddv117)
Supplement: Supplementary Data [file supp_24_13_3732__index.html]

Germline recessive mutations in PI4KA are associated with perisylvian polymicrogyria, cerebellar hypoplasia and arthrogryposis — Germline recessive mutations in PI4KA are associated with perisylvian polymicrogyria, cerebellar hypoplasia and arthrogryposis — Germline recessive mutations in PI4KA are associated with perisylvian polymicrogyria, cerebellar hypoplasia and arthrogryposis — Supplementary Data 

# Germline recessive mutations in *PI4KA* are associated with perisylvian polymicrogyria, cerebellar hypoplasia and arthrogryposis

## Supplementary Data

Supplementary Data

**Files in this Data Supplement:**

- Supplementary Figure 2 - pdf file
- Supplementary Table 1\_Figure 1 - xls file
- Supplementary Table 2 - xls file
