# Supplementary material for: Germline recessive mutations in PI4KA are associated with perisylvian polymicrogyria, cerebellar hypoplasia and arthrogryposis
Source: Hum Mol Genet. 2015 Apr 8;24(13):3732–41. doi: 10.1093/hmg/ddv117 (PMC4459391; doi:10.1093/hmg/ddv117)
Supplement: Supplementary Data [file supp_ddv117_ddv117supp_fig2.pdf]

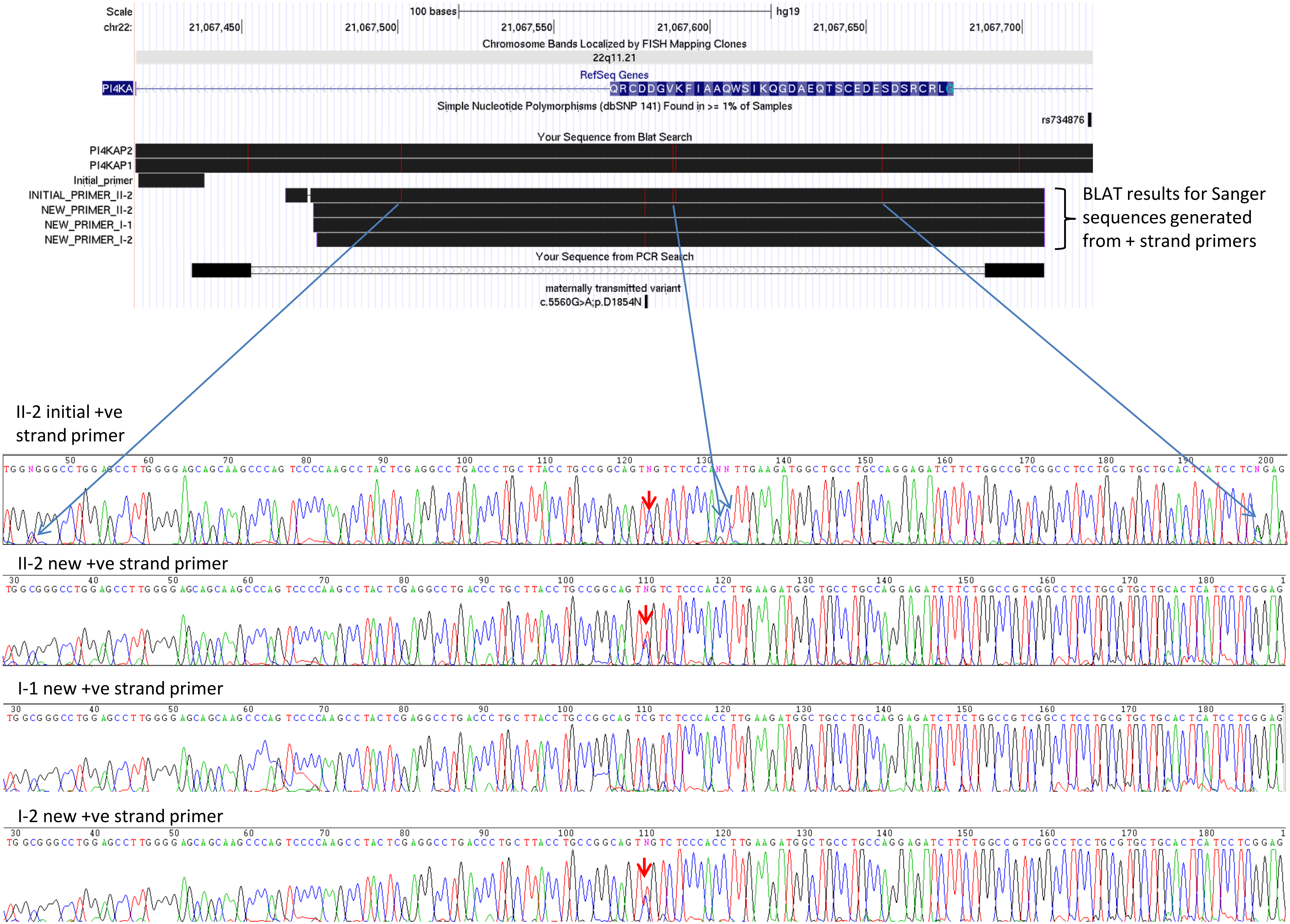

Figure S2: Sanger data generated using new primer aligned to UCSC browser. Blue arrows indicate 4 base positions which are different in the two partial pseudogene copies. Although the initial primer was not completely specific for *PI4KA*, monitoring these 4 positions shows that amplification using the new primers was much more specific. The c.5560G>A variant (appearing here as a C>T on the +ve genomic strand), shown by a red arrow also becomes clearer with the new primer.
